# Association  of Cardiovascular Magnetic Resonance-Derived Papillary Muscle Abnormalities with Non-Sustained Ventricular Tachycardia in Hypertrophic Cardiomyopathy

**DOI:** 10.3390/diagnostics16162599

**Published:** 2026-08-17

**Authors:** Barış Güven, Furkan M. Deniz, Fatih Özkan, Aybüke N. Geylan, Ümit Y. Sinan, Tuba Selcuk Can, Veysel Oktay

**Affiliations:** 1 Department of Cardiology, Liv Hospital Ulus, Istanbul 34340, Turkey; 2 Department of Cardiology, Bagcilar Training and Research Hospital, Istanbul 34200, Turkey; drfurkandeniz@gmail.com; 3 Department of Cardiology, Gaziosmanpasa Training and Research Hospital, Istanbul 34255, Turkey; fatihozkan.fo@gmail.com; 4 Department of Cardiology, Istanbul University-Cerrahpasa Institute of Cardiology, Istanbul 34098, Turkey; aybukegeylan@gmail.com (A.N.G.); drumityasar@hotmail.com (Ü.Y.S.); drvoktay@gmail.com (V.O.); 5 Department of Radiology, Haseki Training and Research Hospital, Istanbul 34096, Turkey; drtubas@gmail.com

**Keywords:** cardiovascular magnetic resonance, hypertrophic cardiomyopathy, late gadolinium enhancement, mitral apparatus, non-sustained ventricular tachycardia, papillary muscle, ventricular arrhythmias

## Abstract

**Background:** Risk stratification for ventricular arrhythmias in hypertrophic cardiomyopathy (HCM) remains challenging, particularly in patients who do not fulfill conventional high-risk criteria. This study aimed to investigate the association between cardiovascular magnetic resonance (CMR)-derived phenotypic characteristics and non-sustained ventricular tachycardia (NSVT) in patients with HCM. **Methods:** A total of 69 consecutive patients with HCM who underwent comprehensive CMR imaging and 24 h Holter monitoring were retrospectively analyzed. CMR assessment included phenotypic characteristics of the left ventricle, mitral valve apparatus, and papillary muscles, as well as late gadolinium enhancement (LGE). Ventricular arrhythmic events were defined as the presence of a premature ventricular complex burden >10%, NSVT, and/or sustained ventricular tachycardia. Univariable logistic regression and multivariable Firth penalized logistic regression analyses were performed to identify variables associated with NSVT. **Results:** The study population had a mean age of 57.5 years, and 23 patients (33.3%) were female. Ventricular arrhythmic events were detected in 16 patients (23.1%), all of whom had NSVT. Median LGE burden was 4%. LGE was present in 43 patients (62.3%), including papillary muscle (PM)-LGE in 13 patients (19.0%). Anterolateral PM–septum systolic contact was observed in 7 patients (10.0%). In univariable analysis, maximal left ventricular wall thickness (OR 1.29, 95% CI 1.09–1.53; *p* = 0.004), abnormal chordal attachment (OR 5.56, 95% CI 1.09–28.19; *p* = 0.039), anterolateral PM mobility (OR 1.45 per 0.1-unit increase, 95% CI 1.10–1.91; *p* = 0.009), anterolateral PM–septum systolic contact (OR 31.20, 95% CI 3.38–288.0; *p* = 0.002), and PM-LGE (OR 9.60, 95% CI 2.50–36.84; *p* < 0.001) were associated with NSVT. In the four-variable Firth penalized logistic regression model, abnormal chordal attachment (OR 9.09, 95% CI 1.18–67.42; *p* = 0.036), anterolateral PM–septum systolic contact (OR 23.08, 95% CI 2.18–385.17; *p* = 0.008), and PM-LGE (OR 17.26, 95% CI 2.37–176.64; *p* = 0.004) remained associated with NSVT. Given the limited number of events, these multivariable findings should be considered exploratory. **Conclusions:** CMR-derived PM abnormalities were associated with NSVT in patients with HCM. In particular, PM-LGE, abnormal chordal attachment, and anterolateral PM–septum systolic contact emerged as exploratory CMR characteristics that warrant further evaluation in larger prospective studies.

## 1. Introduction

Hypertrophic cardiomyopathy (HCM) is one of the most common inherited cardiac diseases [1]. While it may follow an asymptomatic course, it can also present with a broad clinical spectrum, including heart failure, myocardial ischemia, syncope, arrhythmias, and sudden cardiac death (SCD). Among these, SCD secondary to ventricular arrhythmias represents one of the leading causes of mortality in this population [2].

The annual incidence of SCD or appropriate implantable cardioverter-defibrillator therapy due to life-threatening ventricular arrhythmias is approximately 0.8%, a risk that varies considerably across individual risk profiles [3]. Current risk stratification for SCD is based on a multiparametric approach integrating clinical, electrocardiographic, and imaging findings. The ESC HCM Risk-SCD calculator incorporates variables such as age, maximal left ventricular wall thickness, left atrial diameter, left ventricular outflow tract (LVOT) gradient, family history of SCD, unexplained syncope, and non-sustained ventricular tachycardia (NSVT), whereas contemporary ESC and AHA/ACC guidelines additionally recognize the role of cardiovascular magnetic resonance (CMR), particularly for the assessment of left ventricular ejection fraction, apical aneurysm, and late gadolinium enhancement (LGE) [4,5,6,7,8]. In addition, abnormalities of the mitral valve apparatus—including elongated leaflets, anomalous chordal attachments, and papillary muscle (PM) abnormalities—are common phenotypic features of HCM and have been implicated in LVOT obstruction. However, their potential contribution to ventricular arrhythmogenesis remains incompletely understood [9,10,11,12,13,14,15].

CMR imaging plays a pivotal role in the phenotypic characterization of HCM due to its high spatial and temporal resolution. Although echocardiography and CMR are complementary imaging modalities, CMR offers important advantages for detailed phenotypic characterization [16,17,18,19]. It allows a more accurate visualization of the left ventricular apex and more precise assessment of myocardial wall thickness [9,10,20]. In addition, CMR enables a detailed assessment of phenotypic alterations in the mitral valve apparatus and papillary muscles that may contribute to LVOT obstruction [11]. Importantly, CMR is indispensable for the detection and quantification of myocardial fibrosis through LGE imaging [7,9].

Given the highly heterogeneous phenotypic presentation of HCM, further characterization of structural and functional features associated with ventricular arrhythmias may improve our understanding of the arrhythmic phenotype. In this context, the present study aims to identify CMR-derived phenotypic characteristics associated with NSVT in patients with HCM.

## 2. Materials and Methods

### 2.1. Study Population

**Figure 1:**
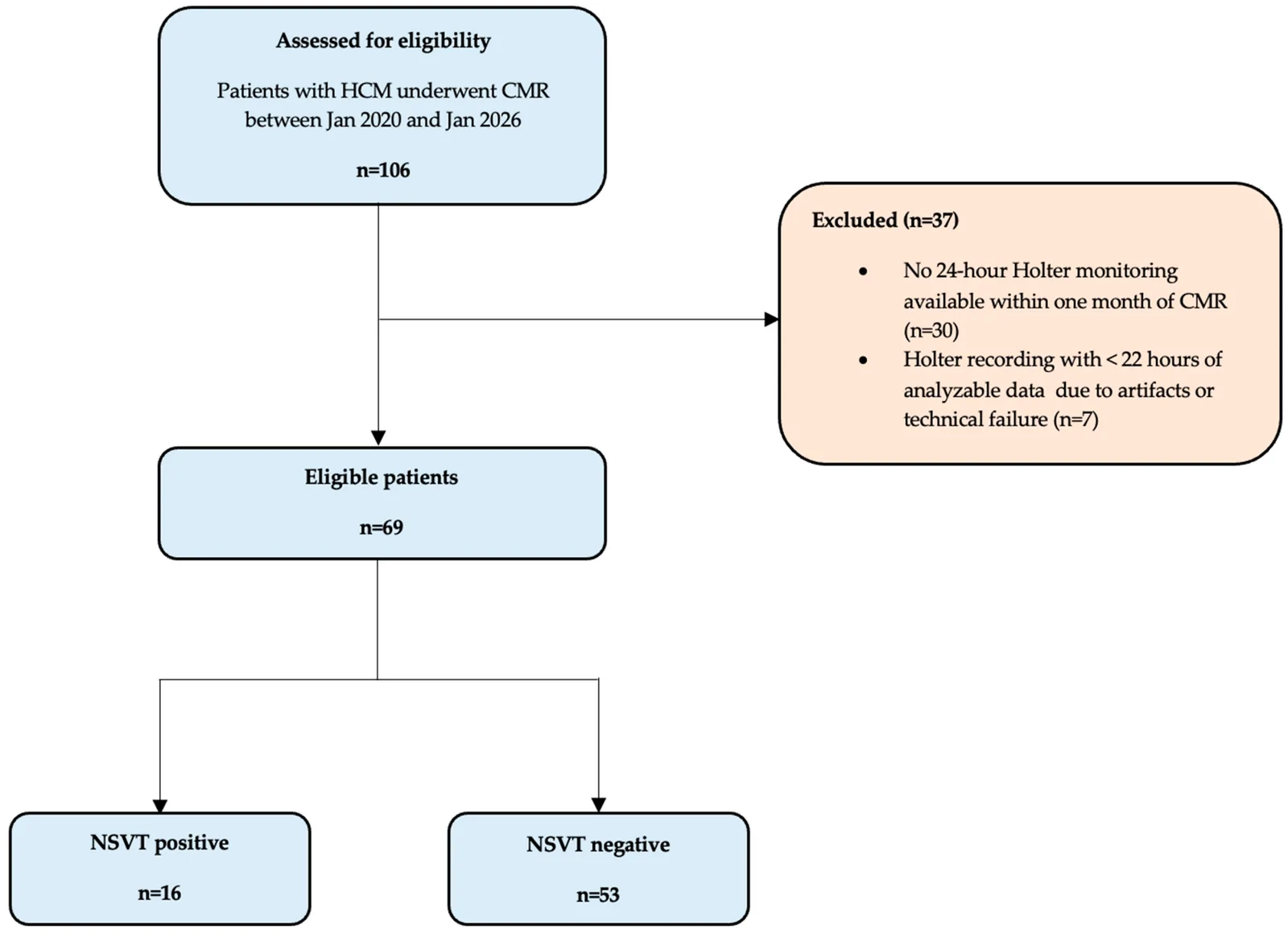
Patient selection flow diagram.

This single-center, cross-sectional study included 106 patients with clinically confirmed HCM based on CMR findings between January 2020 and January 2026. Of these, 30 patients without 24 h Holter monitoring within one month following CMR and 7 patients with <22 h of analyzable Holter recording because of artifacts or technical failure were excluded. Consequently, the final study population consisted of 69 patients (Figure 1).

Eligibility criteria were as follows: (1) age ≥ 18 years; (2) maximal end-diastolic left ventricular wall thickness ≥ 15 mm at any myocardial segment, or a diagnostic threshold of 13–14 mm in the presence of a family history of HCM or LVOT obstruction; (3) no history of prior cardiac surgery or alcohol septal ablation; (4) absence of previously documented significant coronary artery disease or prior myocardial infarction; (5) no more than mild aortic stenosis; and (6) absence of uncontrolled hypertension. Individuals with a diagnosis of, or suspicion for, secondary hypertrophic etiologies other than HCM based on CMR findings or clinical evaluation were not included in the study. Coronary artery disease was not systematically evaluated. Available information was obtained from patients’ medical records and prior coronary angiography reports, when available, without additional investigations performed for study purposes.

LVOT gradient was assessed by continuous-wave Doppler echocardiography. Patients with a gradient ≥30 mmHg at rest or during provocation were classified as having obstructive HCM, whereas the remaining patients were categorized as having non-obstructive HCM.

### 2.2. CMR and Holter Monitoring

CMR imaging was performed using a 1.5-T system (Achieva, Philips Medical Systems, Best, The Netherlands) with ECG gating and respiratory navigation. All patients were scanned in the supine position after standard preparation and breath-hold instruction.

Initial scout images were acquired in axial, sagittal, and coronal planes. Thoracic anatomical assessment was performed using half-Fourier single-shot turbo spin-echo sequences. Cine images were obtained with steady-state free precession sequences in standard long-axis and short-axis views for functional evaluation.

LGE imaging was performed approximately 10 min after intravenous administration of 0.15 mmol/kg gadolinium, using phase-sensitive inversion recovery sequences with inversion time adjusted to null normal myocardium.

Maximal left ventricular wall thickness (MLVWT) and PM morphology were assessed on end-diastolic short-axis cine images. Anterolateral PM mobility was assessed from cine CMR by measuring the shortest distance between the anterolateral PM and the interventricular septum at end-diastole and end-systole. The mobility index was calculated as: (end-diastolic PM–septum distance − end-systolic PM–septum distance)/end-diastolic PM–septum distance. Higher values therefore indicate greater systolic excursion of the PM toward the septum. Anterolateral PM–septum systolic contact was defined as the direct apposition of the anterolateral PM to the interventricular septum during systole, with no visible intervening blood-pool space on short-axis cine images. Only the anterolateral PM was evaluated because the posteromedial PM could not be consistently assessed on the routine CMR cine images available for all patients. Mitral leaflet lengths were measured in three-chamber views, and LVOT diameter was assessed during mid-systole.

Additional structural features, including bifid PM, abnormal chordal attachment, and accessory muscle bundles, were identified based on predefined anatomical criteria [11]. LV apical aneurysm was defined as a thin-walled akinetic or dyskinetic segment.

LGE was defined as myocardial regions with signal intensity >6 standard deviations above the mean signal intensity of reference normal myocardium. LGE extent was quantified by manual planimetry and expressed as a percentage of total LV mass. PM-LGE was assessed separately by a visual evaluation of the papillary muscles on postcontrast PSIR images. Focal hyperenhancement was considered PM-LGE when it anatomically corresponded to the papillary muscle, could be distinguished from the adjacent blood-pool signal, and was confirmed in multiple imaging planes. Suspected PM-LGE was cross-checked on short-axis and long-axis (three- and four-chamber) images to confirm its localization within the PM and minimize misclassification related to blood-pool signal and partial-volume effects. PM-LGE was assessed as a separate binary variable and was not included in the calculation of total LV LGE burden.

Holter monitoring was performed using a standard three-channel device. Each patient underwent a single 24 h Holter recording. Recordings with <22 h of analyzable data because of artifacts or technical failure were considered non-evaluable and were excluded from the analysis. All Holter recordings were analyzed by experienced cardiologists who were blinded to the patients’ clinical information and CMR findings. Standard parameters, including heart rate metrics and arrhythmic burden, were assessed. Ventricular arrhythmias were classified according to standard definitions, including premature ventricular complex (PVC), NSVT (≥3 consecutive beats at a rate > 100 bpm lasting <30 s), and sustained ventricular tachycardia (≥30 s or requiring termination due to hemodynamic instability). The presence of a ventricular arrhythmic event was defined as a PVC burden >10% or the occurrence of NSVT or sustained ventricular tachycardia. A single episode meeting the predefined NSVT criteria was sufficient to classify a patient as NSVT-positive.

All 24 h Holter recordings were obtained within one month following the CMR examination. All CMR studies were analyzed by a single expert cardiovascular radiologist with dedicated experience in CMR who was blinded to the patients’ clinical information and Holter monitoring results.

### 2.3. Statistical Analysis

Statistical analyses were conducted using the Statistical Package for Social Sciences (SPSS) version 23.0 (IBM Corp., Armonk, NY, USA). The Kolmogorov–Smirnov test was used to assess the normality of continuous variables. Normally distributed variables were presented as mean ± standard deviation (SD). Non-normally distributed variables were presented as median and interquartile range (25th–75th percentile). Categorical variables were presented as frequencies and percentages. Univariable logistic regression analyses were performed to explore associations between candidate variables and NSVT. Pairwise relationships among PM variables were assessed using Spearman correlation coefficients, while associations between binary variables were additionally evaluated using contingency tables and Fisher’s exact test. Given the limited number of NSVT events, a parsimonious multivariable model was constructed to reduce model complexity and avoid the simultaneous inclusion of closely related PM variables. The final Firth penalized logistic regression model included four variables: MLVWT, abnormal chordal attachment, anterolateral papillary muscle–septum systolic contact, and PM-LGE. MLVWT was retained as an established conventional morphologic risk marker in HCM, whereas abnormal chordal attachment, papillary muscle–septum systolic contact, and PM-LGE represent distinct structural, mechanical, and fibrotic characteristics relevant to the primary hypothesis of the study. Anterolateral PM mobility was not included in the final multivariable model because pairwise analysis demonstrated substantial overlap with papillary muscle–septum systolic contact, suggesting that these variables capture closely related aspects of the same mechanical phenomenon. Firth penalization was applied to the final multivariable logistic regression model to reduce small-sample bias and potential separation. The analysis was performed using R version 4.5.1 (R Foundation for Statistical Computing, Vienna, Austria) with the logistf package (version 1.26.1). As an internal assessment of model stability, nonparametric bootstrap resampling with 1000 iterations was performed for the final four-variable Firth model. Bootstrap distributions of the regression coefficients were examined to assess the consistency of the direction and magnitude of the estimated associations. There were no missing data for the variables included in the final statistical analyses; therefore, no imputation or other missing data management methods were required. A two-sided *p* value < 0.05 was considered statistically significant. Given the exploratory nature of the study, no adjustment for multiple comparisons was applied; therefore, statistically significant findings should be interpreted as exploratory rather than confirmatory.

Ethical approval was obtained from Istanbul University-Cerrahpasa Medical Research Ethics Committee (decision date: 2 June 2026; approval number: 01). The study protocol was conducted in accordance with the ethical principles outlined in the Declaration of Helsinki. The ethics approval authorized the retrospective review of all medical records included in the study cohort. Informed patient consent was waived because the study involved a retrospective review of existing medical records, in accordance with the requirements of the Institutional Ethics Committee.

## 3. Results

The baseline demographic and clinical characteristics of the study population are summarized in Table 1. The study included 69 patients with HCM, of whom 23 (33.3%) were female. The mean age was 57.5 ± 13.2 years, and the mean body mass index was 29.4 ± 4.2 kg/m^2^. Overall, cardiovascular comorbidities were relatively infrequent, with hypertension and paroxysmal atrial fibrillation being the most common. A family history of SCD and prior syncope were uncommon.

Baseline medical therapy is presented in Table 2**.** Beta-blockers were the predominant treatment (72.5%), whereas calcium channel blockers and disopyramide were used infrequently. None of the patients received treatment with cardiac myosin inhibitors.

CMR findings are summarized in Table 3. Obstructive HCM was present in 24 patients (34.8%), in whom the mean peak LVOT gradient was 78.07 ± 39.41 mmHg. Left ventricular systolic function was generally preserved, with a mean LVEF of 59.8 ± 8.8%, while the mean LV mass index was 63.43 ± 15.06 g/m^2^ and the left atrial volume index was 32.8 ± 16.8 mL/m^2^. Mean MLVWT was 17.35 ± 3.90 mm.

Mitral valve and PM abnormalities were common. Residual mitral leaflet tissue was observed in 26 patients (37.7%), systolic anterior motion in 24 (34.8%), and moderate-to-severe mitral regurgitation in 19 (27.5%). Bifid papillary muscles were identified in 9 patients (13.0%), abnormal chordal attachments in 7 (10.0%), accessory muscle bundles in 4 (5.8%), and anterolateral papillary muscle–septum systolic contact in 7 (10.0%).

LGE was detected in 43 patients (62.3%). LGE involving the right ventricular insertion points was observed in 19 patients (27.5%), while PM-LGE was present in 13 patients (19.0%). The median total LGE burden was 4%.

Twenty-four-hour Holter monitoring findings are presented in Table 4. Sinus rhythm was the predominant rhythm in 66 patients (95.7%), with a mean heart rate of 70 beats per minute. PVC burden exceeding 5% was observed in 3 patients, whereas no patient had a PVC burden exceeding 10%. NSVT was detected in 16 patients (23.1%). No sustained ventricular tachycardia was observed in any patient. Consequently, all patients fulfilling the predefined ventricular arrhythmic endpoint had NSVT.

**Figure 2:**
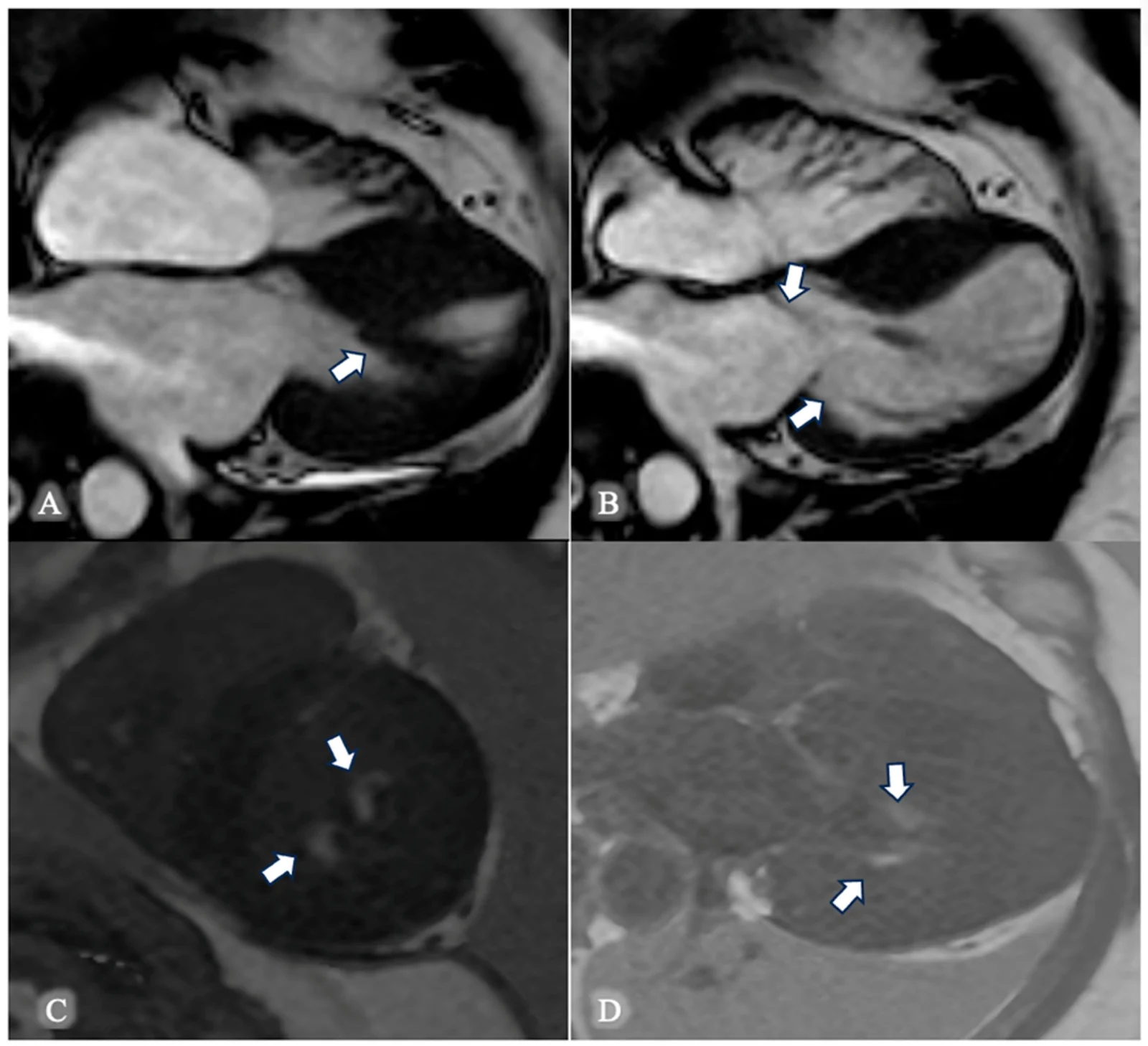
Representative CMR findings of papillary muscle abnormalities. (**A**) Cine SSFP image demonstrating anterolateral papillary muscle–septum systolic contact (arrow). (**B**) Cine SSFP image demonstrating abnormal chordal attachment (arrows). (**C**) Short-axis and (**D**) long-axis postcontrast PSIR images demonstrating focal papillary-muscle late gadolinium enhancement (PM-LGE) (arrows). LGE: late gadolinium enhancement; PM: papillary muscle; PSIR: phase-sensitive inversion recovery; SSFP: steady-state free precession.

Among the binary imaging findings, abnormal chordal attachment was present in 4/16 (25.0%) patients with NSVT and 3/53 (5.7%) without NSVT, anterolateral PM–septum systolic contact in 6/16 (37.5%) and 1/53 (1.9%), respectively, and PM-LGE in 9/16 (56.3%) and 4/53 (7.5%), respectively (Figure 2). Univariable logistic regression analysis demonstrated significant associations between NSVT and maximal left ventricular wall thickness, abnormal chordal attachment, anterolateral PM mobility, anterolateral papillary muscle–septum systolic contact, and PM-LGE (Table 5). Pairwise analysis demonstrated the strongest relationship between anterolateral PM mobility and PM–septum systolic contact (Spearman ρ = 0.515, *p* < 0.001); notably, all seven patients with PM–septum systolic contact had a mobility index of 1.0. Mobility was also correlated with PM-LGE (ρ = 0.321, *p* = 0.007), but not significantly with abnormal chordal attachment (ρ = 0.232, *p* = 0.055). Among the binary variables, PM–septum systolic contact was associated with PM-LGE (Fisher’s exact *p* = 0.020), whereas no significant associations were observed between PM–septum systolic contact and abnormal chordal attachment (*p* = 0.145) or between abnormal chordal attachment and PM-LGE (*p* = 0.609). Given the substantial overlap between mobility and PM–septum systolic contact, mobility was excluded to avoid simultaneous inclusion of closely related variables. The final multivariable model therefore included MLVWT, abnormal chordal attachment, anterolateral PM–septum systolic contact, and PM-LGE. In the four-variable Firth penalized logistic regression model, abnormal chordal attachment (OR 9.09, 95% CI 1.18–67.42; *p* = 0.036), anterolateral papillary muscle–septum systolic contact (OR 23.08, 95% CI 2.18–385.17; *p* = 0.008), and PM-LGE (OR 17.26, 95% CI 2.37–176.64; *p* = 0.004) were associated with NSVT, whereas MLVWT was not statistically significant (OR 0.98, 95% CI 0.76–1.23; *p* = 0.838) (Table 5).

Given the limited number of outcome events and the wide confidence intervals, these multivariable findings should be regarded as exploratory. In the bootstrap stability analysis, the direction of association was preserved in 94.8% of resamples for abnormal chordal attachment, 98.6% for anterolateral papillary muscle–septum systolic contact, and 100% for PM-LGE. However, substantial variability in effect magnitude remained, particularly for the infrequent binary imaging findings, indicating persistent uncertainty in the precision of the estimates.

## 4. Discussion

Although the predefined ventricular arrhythmic endpoint included PVC burden >10%, NSVT, or sustained ventricular tachycardia, all events observed in the present cohort consisted exclusively of NSVT. Therefore, the reported associations should primarily be interpreted as markers associated with NSVT rather than more definitive clinical outcomes such as sustained ventricular tachycardia or sudden cardiac death. Current guidelines incorporate NSVT as a risk marker for SCD alongside several CMR-derived variables. Parameters such as MLVWT, LVEF, and the presence of apical aneurysm are more accurately assessed by CMR compared to echocardiography, while CMR enables the assessment and quantification of myocardial fibrosis burden [3,4,6].

Although these risk models are effective in predicting SCD related to ventricular arrhythmias, the underlying mechanisms in patients who do not meet these criteria yet still develop ventricular arrhythmias remain incompletely understood. Therefore, the primary aim of this study was to identify CMR-derived phenotypic characteristics associated with NSVT in patients with HCM.

CMR has become an integral component of risk stratification in HCM by enabling a comprehensive assessment of myocardial morphology, ventricular function, and fibrosis. Contemporary guidelines recognize CMR as an essential imaging modality for evaluating established risk markers such as maximal left ventricular wall thickness, left ventricular ejection fraction, apical aneurysm, and LGE, while recent studies have further highlighted the prognostic value of quantitative LGE assessment for predicting ventricular arrhythmias and SCD [7,8,10]. Nevertheless, conventional CMR-based risk assessment has largely focused on ventricular morphology and fibrosis burden, whereas comparatively little attention has been directed toward structural abnormalities of the mitral valve apparatus and papillary muscles despite their potential contribution to arrhythmogenesis [9,11]. Given the large number of candidate variables examined relative to the limited number of NSVT events and the absence of adjustment for multiple comparisons, the observed associations should be interpreted as exploratory signals rather than confirmatory findings. Accordingly, interpretation should consider the magnitude and precision of the effect estimates, as reflected by their confidence intervals, together with biological plausibility, rather than statistical significance alone.

Current risk stratification models heavily emphasize the quantitative extent of LGE, with various thresholds cited as robust predictors of SCD, ranging from ≥15% [7] to a comparatively lower threshold of ≥10% as proposed by recent meta-analytical data [8].

In the present cohort, neither total LGE burden nor right ventricular insertion-point LGE was significantly associated with NSVT in univariable analyses. In contrast, PM-LGE showed a significant association with NSVT in univariable analysis and was also associated with NSVT in the final multivariable model. This finding raises the hypothesis that the spatial distribution of fibrosis may be relevant to arrhythmic risk in HCM, although the present study cannot establish a mechanistic relationship. As suggested by Klopotowski et al., a qualitative assessment of LGE morphology may provide information not fully captured by quantitative LGE burden alone [12]. Our results are consistent with prior observations by Amano et al. and Barbosa et al., who demonstrated that non-septal LGE distributions—particularly involving the papillary muscles—are more strongly associated with ventricular tachyarrhythmias [13,14]. Whether PM fibrosis contributes directly to an arrhythmogenic substrate or simply reflects a broader HCM phenotype cannot be determined from the present study.

In our cohort, the median LGE burden was only 4%, considerably lower than the thresholds (≥10–15%) that have been associated with increased arrhythmic risk in previous studies. This relatively low fibrotic burden may partly reflect the characteristics of our single-center study population and could have attenuated the previously established association between overall LGE burden and ventricular arrhythmias. Therefore, our findings regarding global LGE burden should be interpreted with caution and may not be directly generalizable to HCM populations with more extensive myocardial fibrosis. Nevertheless, despite the relatively low overall fibrotic burden, the association between papillary muscle fibrosis and NSVT suggests that fibrosis localization may represent a potentially relevant marker of the arrhythmic phenotype.

One of the notable findings of this study was the association between anterolateral papillary muscle–septum systolic contact and NSVT. Although this observation is consistent with the proposed “percussion injury” hypothesis, our study was not designed to determine whether repetitive papillary muscle–septum contact directly causes fibrosis or ventricular arrhythmias. The proposed mechanism therefore remains speculative and is based on previously published mechanistic concepts, including the framework described by Opris et al., which suggests that chronic mechanical stress on the subvalvular apparatus may contribute to fibrotic remodeling [15]. Likewise, the observed association between abnormal chordal attachments and NSVT may reflect a contribution of mitral apparatus abnormalities to this process, although causality cannot be inferred from the present cross-sectional study. MLVWT remains a cornerstone of ESC and ACC risk stratification models. Although MLVWT was significantly associated with NSVT in the univariable analysis, this association was not retained in the final multivariable model and should be interpreted cautiously given the limited number of events and the exploratory nature of the analysis. This observation may be considered in the context of the “inverted U-shaped” relationship described by O’Mahony et al., in which SCD risk increases from mild to moderate hypertrophy before plateauing at extreme wall thicknesses (≥35 mm) [21]. As highlighted by Olivotto et al. and Norrish et al., MLVWT alone may be insufficient as a standalone predictor in patients with non-extreme hypertrophy [22,23]. In patients without extreme hypertrophy, other morphological and fibrotic characteristics, including PM abnormalities, may therefore warrant further investigation in relation to ventricular arrhythmias.

## 5. Limitations

This study has several important limitations. First, its single-center, cross-sectional design, relatively small sample size, and limited number of NSVT events may have affected the statistical precision and generalizability of the findings. No a priori sample size or power calculation was performed. Although Firth penalization and a parsimonious multivariable model were used to mitigate small-sample bias and model instability, the risks of overfitting and model-selection bias remain. In addition, no adjustment for multiple comparisons was performed despite the evaluation of numerous clinical and imaging variables. Therefore, the observed associations should be interpreted based on effect estimates, confidence intervals, and biological plausibility and regarded as exploratory and hypothesis-generating rather than confirmatory.

Second, the absence of sustained ventricular tachycardia and the exclusive occurrence of NSVT may reflect a relatively low-risk and selected cohort. Selection bias related to Holter availability is also possible: 30 of 106 screened patients lacked Holter monitoring within one month of CMR, and 7 had non-evaluable recordings. Because Holter monitoring was performed at the discretion of the treating physician, and the characteristics of excluded patients and indications for monitoring were not systematically available, patients undergoing Holter monitoring may have differed from those without monitoring. Furthermore, NSVT assessment was based on a single 24 h recording, which may have resulted in outcome misclassification because of the intermittent nature of NSVT. Detailed episode-level characteristics, including the number, maximum rate, duration, and number of beats, were not systematically available.

Third, genotype data were not systematically available, limiting comprehensive risk characterization. Coronary artery disease was also not systematically assessed, and the number of patients who underwent formal coronary evaluation could not be reliably determined. Thus, unrecognized coronary artery disease or ischemic scar cannot be completely excluded as a potential confounder of the associations between LGE characteristics and NSVT.

Finally, CMR measurements and qualitative assessments were performed by a single reader, and formal inter- and intra-observer reproducibility analyses were not available. This is particularly relevant to the novel and potentially operator-dependent PM characteristics. Although predefined measurement criteria based on our previously published methodology were applied, their reproducibility requires confirmation in future studies.

## 6. Conclusions

In conclusion, our findings identify CMR-derived PM characteristics, particularly PM-LGE and papillary muscle–septum systolic contact, which were associated with NSVT in patients with HCM. Given the exploratory design and limited number of events, these findings should be considered hypothesis-generating and warrant validation in larger prospective multicenter studies.

## Figures and Tables

**Table 1:** Demographic and clinical characteristics.

Variables	All Patients (*n* = 69)
Female, *n* (%)	23 (33.3%)
Age (year)	57.5 ± 13.2
BMI (kg/m^2^)	29.4 ± 4.2
SBP (mmHg)	130 (120–140)
DBP (mmHg)	80 (70–80)
HT, *n* (%)	23 (33.3)
DM, *n* (%)	15 (21.7)
PAF, *n* (%)	22 (31.9)
CKD, *n* (%)	5 (7.2)
CVE, *n* (%)	4 (5.8)
Family history of HCM, *n* (%)	5 (7.2)
Family history of SCD, *n* (%)	2 (2.9)
Syncope, *n* (%)	4 (5.8)

BMI: body mass index; CKD: chronic kidney disease; CVE: cerebrovascular event; DBP: diastolic blood pressure; DM: diabetes mellitus; HCM: hypertrophic cardiomyopathy; HT: hypertension; PAF: paroxysmal atrial fibrillation; SBP: systolic blood pressure; SCD: sudden cardiac death.

**Table 2:** Medical therapy.

Variables	All Patients (*n* = 69)
Beta-blocker use, *n* (%)	50 (72.5)
Calcium channel blocker use, *n* (%)	4 (5.8)
Disopyramide use, *n* (%)	2 (2.9)
Anticoagulant use, *n* (%)	19 (27.5)
Diuretic use, *n* (%)	21 (30.4)
Cardiac myosin inhibitors use, *n* (%)	0 (0)

**Table 3:** Phenotypic characteristics assessed on CMR.

Variables	All Patients (*n* = 69)
Obstructive HCM *, *n* (%)	24 (34.8)
Maximum LVOT gradient * (mmHg)	78.07 ± 39.41
Ejection fraction (%)	59.80 ± 8.80
EDV index (mL/m^2^)	67.88 ± 19.08
ESV index (mL/m^2^)	27.83 ± 11.65
SV index (mL/m^2^)	39.64 ± 11.36
LV mass index (g/m^2^)	63.43 ± 15.06
LA volume index (mL/m^2^)	32.8 ± 16.8
LASI	0.94 ± 0.34
IVS mid thickness (mm)	14.48 ± 4.52
IVS basal thickness (mm)	13.75 ± 4.33
PW mid thickness (mm)	10.0 (8.0–12.0)
MLVWT (mm)	17.35 ± 3.90
Apical HCM, *n* (%)	3 (4.3)
LVOT diameter (mm)	12.68 ± 3.98
Anterior mitral leaflet length (mm)	26.03 ± 3.16
Posterior mitral leaflet length (mm)	19.26 ± 2.80
Night cap (mm)	9.10 ± 3.39
Residual mitral leaflet, *n* (%)	26 (37.7)
Coaptation zone (mm)	4.0 (3.0–5.0)
SAM, *n* (%)	24 (34.8)
MR, *n* (%) Moderate to severe MR, *n* (%)	39 (56.5) 19 (27.5)
Bifid PM, *n* (%)	9 (13.0)
Total PM head area, mm^2^	112.25 ± 42.81
Abnormal chordal attachment, *n* (%)	7 (10.0)
Anterolateral PM position ratio	0.63 ± 0.10
Posteromedial PM position ratio	0.63 ± 0.09
Accessory muscle bundles, *n* (%)	4 (5.8)
Anterolateral PM–septum distance at systole (mm)	4.44 ± 3.30
Anterolateral PM–septum distance at diastole (mm)	9.83 ± 4.33
Anterolateral PM mobility	0.60 ± 0.22
Anterolateral PM–septum systolic contact, *n* (%)	7 (10.0)
Presence of LGE, *n* (%)	43 (62.3)
RV insertion-point LGE, *n* (%)	19 (27.5)
PM-LGE, *n* (%)	13 (19.0)
LGE burden (%)	4.0 (0–8.0)

* These variables were obtained from echocardiographic assessments. CMR: cardiovascular magnetic resonance; EDV: end-diastolic volume; ESV: end-systolic volume; HCM: hypertrophic cardiomyopathy; IVS: interventricular septum; LA: left atrium; LASI: left atrium sphericity index; LGE: late gadolinium enhancement; LV: left ventricle; LVOT: left ventricular outflow tract; MLVWT: maximal left ventricular wall thickness; MR: mitral regurgitation; PM: papillary muscle; PW: posterior wall; SAM: systolic anterior motion; SV: stroke volume.

**Table 4:** Twenty-four-hour Holter monitoring findings.

Variables	All Patients (*n* = 69)
Sinus rhythm, *n* (%)	66 (95.7)
Mean heart rate (beat/min)	70.03 ± 9.40
Total beat count (beat/day)	96 720 ± 12 917
PVC burden > 5%, *n* (%)	3 (4.3)
PVC burden > 10%, *n* (%)	0
NSVT, *n* (%)	16 (23.1)
Sustained ventricular tachycardia, *n* (%)	0
Ventricular arrhythmic event, *n* (%)	16 (23.1)

NSVT: non-sustained ventricular tachycardia; PVC: premature ventricular complex.

**Table 5:** Univariable logistic regression and multivariable Firth penalized logistic regression analyses of variables associated with NSVT.

	Univariable Analysis		Multivariable Analysis	
Variables	OR (95% CI)	*p* Value	OR (95% CI)	*p* Value
Female	1.27 (0.40–4.07)	*p* = 0.687		
Age	1.03 (0.98–1.07)	*p* = 0.245		
BMI	0.874 (0.73–1.04)	*p* = 0.125		
SBP	0.98 (0.93–1.02)	*p* = 0.334		
DBP	0.98 (0.92–1.05)	*p* = 0.658		
HT	0.88 (0.27–2.94)	*p* = 0.840		
DM	0.19 (0.02–1.54)	*p* = 0.119		
PAF	1.39 (0.43–4.47)	*p* = 0.583		
CKD	0.82 (0.08–7.88)	*p* = 0.861		
CVE	1.11 (0.11–11.5)	*p* = 0.930		
Family history of HCM	2.38 (0.36–15.68)	*p* = 0.367		
Family history of SCD	3.47 (0.20–58.77)	*p* = 0.389		
Syncope	1.11 (0.11–11.48)	*p* = 0.930		
Beta-blocker use	7.71 (0.94–63.16)	*p* = 0.057		
Anticoagulant use	1.27 (0.37–4.29)	*p* = 0.705		
Diuretic use	0.26 (0.05–1.25)	*p* = 0.092		
Obstructive HCM *	0.81 (0.25–2.69)	*p* = 0.735		
Max LVOT gradient *	0.99 (0.98–1.01)	*p* = 0.669		
Ejection fraction	0.97 (0.91–1.04)	*p* = 0.368		
EDV index	0.99 (0.96–1.03)	*p* = 0.651		
ESV index	1.01 (0.96–1.07)	*p* = 0.649		
SV index	0.96 (0.91–1.02)	*p* = 0.196		
LV mass index	1.02 (0.97–1.06)	*p* = 0.470		
LA volume index	1.033 (0.996–1.071)	*p* = 0.080		
LASI	0.91 (0.18–4.77)	*p* = 0.916		
IVS mid thickness	1.12 (0.99–1.27)	*p* = 0.070		
IVS basal thickness	1.04 (0.91–1.18)	*p* = 0.571		
PW mid thickness	0.97 (0.81–1.16)	*p* = 0.734		
MLVWT	1.29 (1.09–1.53)	***p*** ** = 0.004**	0.98 (0.76–1.23)	*p* = 0.838
LVOT diameter	1.01 (0.88–1.16)	*p* = 0.879		
Anterior mitral leaflet length	1.02 (0.85–1.22)	*p* = 0.818		
Posterior mitral leaflet length	0.90 (0.73–1.10)	*p* = 0.299		
Night cap	0.97 (0.82–1.15)	*p* = 0.759		
Residual mitral leaflet	0.47 (0.13–1.65)	*p* = 0.296		
Coaptation zone	0.95 (0.66–1.37)	*p* = 0.816		
SAM	0.81 (0.25–2.69)	*p* = 0.735		
MR	1.38 (0.44–4.35)	*p* = 0.538		
Moderate to severe MR	0.84 (0.23–3.04)	*p* = 0.796		
Bifid PM	1.80 (0.40–8.23)	*p* = 0.444		
Total PM head area, mm^2^	0.99 (0.98–1.01)	*p* = 0.475		
Abnormal chordal attachment, *n* (%)	5.56 (1.09–28.19)	***p*** ** = 0.039**	9.09 (1.18–67.42)	***p*** ** = 0.036**
Anterolateral PM position ratio	0.21 (0.0006–4.26)	*p* = 0.597		
Posteromedial PM position ratio	0.35 (0.0006–5.27)	*p* = 0.747		
Accessory muscle bundles, *n* (%)	1.11 (0.11–11.48)	*p* = 0.930		
Anterolateral PM mobility (per 0.1-unit increase)	1.45 (1.10–1.91)	***p*** ** = 0.009**		
Anterolateral PM–septum systolic contact, *n* (%)	31.20 (3.38–288.0)	***p*** ** = 0.002**	23.08 (2.18–385.17)	***p*** ** = 0.008**
Presence of LGE	2.13 (0.61–7.48)	*p* = 0.239		
RV insertion-point LGE	1.85 (0.56–6.07)	*p* = 0.313		
PM-LGE	9.60 (2.50–36.84)	***p*** ** < 0.001**	17.26 (2.37–176.64)	***p*** ** = 0.004**
LGE burden	1.01 (0.95–1.08)	*p* = 0.656		

* These variables were obtained from echocardiographic assessments. BMI: body mass index; CKD: chronic kidney disease; CVE: cerebrovascular event; DBP: diastolic blood pressure; DM: diabetes mellitus; EDV: end-diastolic volume; ESV: end-systolic volume; HCM: hypertrophic cardiomyopathy; HT: hypertension; IVS: interventricular septum; LA: left atrium; LASI: left atrium sphericity index; LGE: late gadolinium enhancement; LV: left ventricle; LVOT: left ventricular outflow tract; MLVWT: maximal left ventricular wall thickness; MR: mitral regurgitation; PAF: paroxysmal atrial fibrillation; PM: papillary muscle; PW: posterior wall; SAM: systolic anterior motion; SBP: systolic blood pressure; SCD: sudden cardiac death; SV: stroke volume; Bold values indicate statistical significance (*p* < 0.05).

## Data Availability

The original contributions presented in this study are included in the article. Further inquiries can be directed to the corresponding author.

## References

[1] Burns J., Jean-Pierre P. (2018). Disparities in the Diagnosis of Hypertrophic Obstructive Cardiomyopathy: A Narrative Review of Current Literature. Cardiol. Res. Pract..

[2] Tower-Rader A., Desai M., Griffin B., Menon V. (2018). Hypertrophic Cardiomyopathy. Manual of Cardiovascular Medicine.

[3] Zeppenfeld K., Tfelt-Hansen J., de Riva M., Winkel B.G., Behr E.R., A Blom N., Charron P., Corrado D., Dagres N., de Chillou C. (2022). 2022 ESC Guidelines for the management of patients with ventricular arrhythmias and the prevention of sudden cardiac death. Eur. Heart J..

[4] Arbelo E., Protonotarios A., Gimeno J.R., Arbustini E., Barriales-Villa R., Basso C., Bezzina C.R., Biagini E., A Blom N., A de Boer R. (2023). 2023 ESC Guidelines for the management of cardiomyopathies: Developed by the task force on the management of cardiomyopathies of the European So-ciety of Cardiology (ESC). Eur. Heart J..

[5] Ommen S.R., Mital S., Burke M.A., Day S.M., Deswal A., Elliott P., Evanovich L.L., Hung J., Joglar J.A., Kantor P. (2020). 2020 AHA/ACC Guideline for the Diagnosis and Treatment of Patients with Hypertrophic Cardiomyopathy. J. Am. Coll. Cardiol..

[6] Ommen S.R., Ho C.Y., Asif I.M., Balaji S., Burke M.A., Day S.M., Dearani J.A., Epps K.C., Evanovich L., Ferrari V.A. (2024). 2024 AHA/ACC/AMSSM/HRS/PACES/SCMR Guideline for the Management of Hypertrophic Cardiomyopathy: A Report of the American Heart Association/American College of Cardiology Joint Committee on Clinical Practice Guidelines. Circulation.

[7] Chan R.H., Maron B.J., Olivotto I., Pencina M.J., Assenza G.E., Haas T., Lesser J.R., Gruner C., Crean A.M., Rakowski H. (2014). Prognostic Value of Quantitative Contrast-Enhanced Cardiovascular Magnetic Resonance for the Evaluation of Sudden Death Risk in Patients with Hypertrophic Cardiomyopathy. Circulation.

[8] Kiaos A., Daskalopoulos G.N., Kamperidis V., Ziakas A., Efthimiadis G., Karamitsos T.D. (2024). Quantitative Late Gadolinium Enhancement Cardiac Magnetic Resonance and Sudden Death in Hypertrophic Cardiomyopathy. JACC Cardiovasc. Imaging.

[9] Rowin E.J., Maron B.J., Maron M.S. (2020). The Hypertrophic Cardiomyopathy Phenotype Viewed Through the Prism of Multimodality Imaging. JACC Cardiovasc. Imaging.

[10] Maron B.J., Desai M.Y., Nishimura R.A., Spirito P., Rakowski H., Towbin J.A., Rowin E.J., Maron M.S., Sherrid M.V. (2022). Diagnosis and Evaluation of Hypertrophic Cardiomyopathy. J. Am. Coll. Cardiol..

[11] Güven B., Can T.S., Deniz M.F., Geçit M.H., Geylan N.A., Sinan Ü.Y., Oktay V., Ersanlı M.K. (2024). Evaluation of potential links between phenotypic features and genetic variants in left ventricular outflow tract obstruction in hypertrophic cardiomyopathy using cardiovascular magnetic resonance imaging. Int. J. Cardiovasc. Imaging.

[12] Klopotowski M., Kukula K., Malek L.A., Spiewak M., Polanska-Skrzypczyk M., Jamiolkowski J., Dabrowski M., Baranowski R., Klisiewicz A., Kusmierczyk M. (2016). The value of cardiac magnetic resonance and distribution of late gadolinium enhancement for risk stratification of sudden cardiac death in patients with hypertrophic cardiomyopathy. J. Cardiol..

[13] Barbosa A.R., Almeida J., Guerreiro C., Teixeira P., Lopes R.L., Ferreira N.D., Sousa O., Braga P. (2020). Late gadolinium enhancement location assessed by magnetic resonance and arrhythmogenic risk in hypertrophic cardiomyopathy. Rev. Port. Cardiol..

[14] Amano Y., Yanagisawa F., Kitamura M., Tachi M., Kumita S. (2017). Relationship of Nonseptal Late Gadolinium Enhancement to Ventricular Tachyarrhythmia in Hypertrophic Cardiomyopathy. J. Comput. Assist. Tomogr..

[15] Opris D.R., Harpa M.M., Anitei D.-E., Calburean P., Rudzik R. (2025). Mitral Valve Prolapse and Sudden Cardiac Death—A Puzzle with Missing Pieces: Review of the Literature and Case Report. Med. Sci..

[16] Bitar R., Leung G., Perng R., Tadros S., Moody A.R., Sarrazin J., McGregor C., Christakis M., Symons S., Nelson A. (2006). MR Pulse Sequences: What Every Radiologist Wants to Know but Is Afraid to Ask. RadioGraphics.

[17] Gaba R.C., Carlos R.C., Weadock W.J., Reddy G.P., Sneider M.B., Cascade P.N. (2002). Cardiovascular MR Imaging: Technique Optimization and Detection of Disease in Clinical Practice. RadioGraphics.

[18] Ginat D.T., Fong M.W., Tuttle D.J., Hobbs S.K., Vyas R.C. (2011). Cardiac Imaging: Part 1, MR Pulse Sequences, Imaging Planes, and Basic Anatomy. Am. J. Roentgenol..

[19] Paksoy Y., Sivri M. (2019). Kardiyak MRG Teknikleri. Türk Radyoloji Semin..

[20] Maron B.J. (2018). Clinical Course and Management of Hypertrophic Cardiomyopathy. N. Engl. J. Med..

[21] O’Mahony C., Jichi F., Pavlou M., Monserrat L., Anastasakis A., Rapezzi C., Biagini E., Gimeno J.R., Limongelli G., McKenna W.J. (2014). A novel clinical risk prediction model for sudden cardiac death in hypertrophic cardiomyopathy (HCM Risk-SCD). Eur. Heart J..

[22] Norrish G., Ding T., Field E., Cervi E., Ziółkowska L., Olivotto I., Khraiche D., Limongelli G., Anastasakis A., Weintraub R. (2022). Relationship Between Maximal Left Ventricular Wall Thickness and Sudden Cardiac Death in Childhood Onset Hypertrophic Cardiomyopathy. Circ. Arrhythmia Electrophysiol..

[23] Olivotto I., Gistri R., Petrone P., Pedemonte E., Vargiu D., Cecchi F. (2003). Maximum left ventricular thickness and risk of sudden death in patients with hypertrophic cardiomyopathy. J. Am. Coll. Cardiol..

